# Supplementary material for: Cloud BioLinux: pre-configured and on-demand bioinformatics computing for the genomics community
Source: BMC Bioinformatics. 2012 Mar 19;13:42. doi: 10.1186/1471-2105-13-42 (PMC3372431; doi:10.1186/1471-2105-13-42)
Supplement: Additional file 1 — Supplementary 1 Cloud BioLinux software documentation in the form of a mini, self-contained website. Users need to download and uncompress the .zip file, and open through a web browser the "index.html" file available on the main directory. (ZIP 1823 kb). [file 1471-2105-13-42-S1.ZIP › Cloud-BioLinux-Package-Documentation/docs/swat.html]

Bio-Linux Software Documentation Pages

Back to search form

## swat

|  |  |
| --- | --- |
| Name | swat |
| Description | **swat** is a program for searching one or more DNA or protein query sequences, or a query profile, against a sequence database, using an efficient implementation of the Smith-Waterman or Needleman-Wunsch algorithms with linear (affine) gap penalties. For each match an empirical measure of statistical significance derived from the observed score distribution is computed.  **Swat** is ***licensed software*** and thus is ***not*** installed by default on Bio-Linux. It is free to academic researchers and comes as part of the phrap package, but you must obtain a license to get the software. Further information on how to obtain phrap/cross\_match/swat is available on the official website for phred, phrap and consed. |
| Homepage | http://www.phrap.org |
| Remote Documentation | http://www.phrap.org/phredphrapconsed.html#block\_phrap   http://www.phrap.org/phredphrap/swat.html |
